# Reproducibility of extracellular vesicle research

**DOI:** 10.1002/jev2.70036

**Published:** 2025-01-17

**Authors:** Rossella Crescitelli, Juan Falcon‐Perez, An Hendrix, Metka Lenassi, Le Thi Nguyet Minh, Takahiro Ochiya, Nicole Noren Hooten, Ursula Sandau, Clotilde Théry, Rienk Nieuwland

**Affiliations:** ^1^ Sahlgrenska Center for Cancer Research, Department of Surgery, Institute of Clinical Sciences, Sahlgrenska Academy University of Gothenburg Gothenburg Sweden; ^2^ Exosomes Laboratory, Center for Cooperative Research in Biosciences (CIC bioGUNE) Basque Research and Technology Alliance (BRTA) Derio Spain; ^3^ Metabolomics Platform CIC bioGUNE Derio Spain; ^4^ Centro de Investigación Biomédica en Red de Enfermedades Hepáticas y Digestivas (Ciberehd) Madrid Spain; ^5^ Basque Foundation for Science Ikerbasque Bilbao Spain; ^6^ Laboratory of Experimental Cancer Research, Department of Human Structure and Repair Ghent University Ghent Belgium; ^7^ Cancer Research Institute Ghent Ghent Belgium; ^8^ Institute of Biochemistry and Molecular Genetics, Faculty of Medicine University of Ljubljana Ljubljana Slovenia; ^9^ Department of Pharmacology and Institute for Digital Medicine, Yong Loo Lin School of Medicine National University of Singapore Singapore Singapore; ^10^ Department of Molecular and Cellular Medicine Tokyo Medical University Shinjyuku‐ku Tokyo Japan; ^11^ Laboratory of Epidemiology and Population Sciences National Institute on Aging, National Institutes of Health Baltimore Maryland USA; ^12^ Department of Anesthesiology & Perioperative Medicine Oregon Health & Science University Portland Oregon USA; ^13^ INSERM U932 Institut Curie, PSL Research University Paris France; ^14^ Laboratory of Experimental Clinical Chemistry, Laboratory Specialized Diagnostics & Research, Department of Laboratory Medicine Amsterdam UMC, University of Amsterdam Amsterdam The Netherlands; ^15^ Amsterdam Vesicle Center Amsterdam The Netherlands

1

Ever since its launch in 2011, the International Society for Extracellular Vesicles (ISEV) has endorsed, initiated, and supported original ideas and solutions to promote reproducibility (Hill et al., 2013; Lötvall et al., 2014; Théry et al., 2018; Welsh et al., 2024) and these efforts have been appreciated by the general scientific community (Abbott, 2023). Improving reproducibility is complex and multifactorial, and involves development of protocols, reference materials and standards, interlaboratory comparison studies, instrument calibration, transparent reporting, and education.

To support reproducibility, ISEV founded the Rigor and Standardization (R&S) Subcommittee in 2019, which now includes fifteen task forces and three inter‐societal working groups (https://www.isev.org/rigor‐standardization). Within this subcommittee, hundreds of ISEV members have become actively involved in R&S, and together they are working on creative solutions to overcome the challenges of reproducibility in the EV field.

EV research invariably involves collection, handling, and storage of (EV‐containing) fluids, such as body fluids and conditioned culture media, and tissues, which are the starting materials for EV research. Collection, handling and storage of (purified) EV‐containing materials will affect their composition. For example, preparation of plasma and serum, which are amongst the most commonly used body fluids for EV research (Royo et al., 2020), involves about 40 variables which all may impact the sample composition and downstream analysis of EVs (Clayton et al., 2019). Laboratories and biobanks preparing and storing EV‐containing tissues and fluids, commonly use their own ‘in‐house’ protocols, which all may differ from each other and have unknown effects on the sample composition and downstream analysis of EVs (López‐Guerrero et al., 2023). Moreover, even when the same protocol is used, the sample composition may still vary (Bettin et al., 2022) and these differences can be sufficient to affect the results of downstream EV characterization (Bracht et al., 2023). Taken together, sample preparation and storage invariably leads to variability in sample composition, thereby introducing an ‘uneven playing field’ and bias which can hamper the comparability, interpretation and reproducibility of results on EVs.

At present, the current approach to improve reproducibility is by reporting the protocol of sample preparation in the Materials and Methods section of scientific manuscripts. Unfortunately, this reporting is often incomplete or inconsistent between manuscripts, thereby hampering reproducibility. There can be multiple reasons for incomplete or inconsistent reporting, ranging from researchers not knowing these details themselves, or because the journal has a strict word count, or the details are considered as too basic. Importantly, ISEV supports to upload the detailed preparation protocols in EV‐TRACK, an online and open access database that was founded to improve transparent reporting of EV methodology (Van Deun et al., 2017). Despite reporting, however, still hundreds of different and unique preparation protocols co‐exist with unknown effects on the sample composition and downstream analysis of EVs. Thus, the question arises whether there are alternative solutions that can improve the reproducibility of EV research but without interfering with individual researchers’ freedom.

Recently, the Blood EV task force came up with an innovative solution. They proposed to measure and report the composition of the prepared blood plasma and serum samples, in addition to the protocol that was used for sample preparation (Lucien et al., 2023). This solution has multiple advantages. Firstly, local protocols and infrastructure do not have to be changed, which is especially important for established biobanks and ongoing studies. Secondly, by measuring and transparently reporting the sample composition, relevant information is shared with the scientific community that may be useful for correct interpretation of results. Thirdly, collected information from multiple studies can be stored in a centralized database. In turn, this information can be analysed retrospectively to develop evidence‐based sample inclusion and exclusion criteria (i.e. for certain downstream EV applications), as well as evidence‐based preparation protocols.

There can be valid reasons why the sample composition cannot be measured and reported, for example due to limited amount of available sample, or the lack of infrastructure to measure the sample composition. Importantly, not reporting the sample composition should not prohibit the results from being published, but rather should be considered as a study limitation. The main and overall goal is that by transparently reporting the sample composition, retrospectively the validity and reproducibility of published results can be judged better over time, and in an independent and evidence‐based manner. Importantly, such an approach shifts the focus from ‘rigor and standardization’ to ‘reproducibility’.

Despite the clear mission of the R&S Subcommittee, there have been questions whether its name truly represents its goals. Especially the term ‘standardization’ suggests that researchers are told ‘what to do’, and this term tends to exclude and deter researchers rather than to include them. Therefore, we propose to change the current name of *Rigor and Standardization Subcommittee* into *Scientific Reproducibility (Sub) Committee*. In our view, this new name better reflects the goals of task forces and intersociety working groups than the original name, and emphasises ISEVs’ intention to take reproducibility earnestly.

## AUTHOR CONTRIBUTIONS

Rossella Crescitelli: Writing—review and editing (equal). Juan Falcon‐Perez: Writing—review and editing (equal). An Hendrix: Writing—review and editing (equal). Metka Lenassi: Writing—original draft (equal); writing—review and editing (equal). Le Thi Nguyet Minh: Writing—review and editing (equal). Takahiro Ochiya: Writing—review and editing (equal). Nicole Noren Hooten: Writing—review and editing (equal). Ursula Sandau: Writing—review and editing (equal). Clotilde Théry: Writing—review and editing (equal). Rienk Nieuwland: Conceptualization (lead); writing—original draft (lead); writing—review and editing (equal).

## DISCLOSURES

RC has developed multiple EV‐associated patents for putative clinical utilization: US20200088734A1, United States; WO2020146390A1, WIPO (PCT). RC owns equity in Exocure Bioscience Inc. AH is inventor on patents and/or patent applications related to extracellular vesicle products. MTNL is a cofounder and advisor of Carmine Therapeutics. CT is inventor of two patents on therapeutic use of EVs
